# Clinical and predictive significance of Plasma Fibrinogen Concentrations combined Monocyte-lymphocyte ratio in patients with Diabetic Retinopathy

**DOI:** 10.7150/ijms.51533

**Published:** 2021-01-26

**Authors:** Qinghua Huang, Hui Wu, Mingyi Wo, Jiangbo Ma, Yingxiang Song, Xianming Fei

**Affiliations:** 1 Department of Endocrinology, Zhejiang Provincial People's Hospital, and People's Hospital of Hangzhou Medical College, Hangzhou, Zhejiang, China.; 2 Key Laboratory of Endocrine Gland Diseases of Zhejiang Province, Hangzhou, Zhejiang, China.; 3 Center for Laboratory Medicine, Zhejiang Provincial People's Hospital, and People's Hospital of Hangzhou Medical College, Hangzhou, Zhejiang, China.

**Keywords:** diabetic retinopathy, fibrinogen concentrations, moncyte-lymphocyte ratio, predictor

## Abstract

Diabetic retinopathy (DR) is one of the most common causes of blindness and visual impairment. Therefore, early prediction of its occurrence and progression is important. This study aimed to assess the clinical and predictive significance of plasma fibrinogen concentrations combined monocyte-lymphocyte ratio (FC-MLR) in patients with DR. A total of 307 patients with type 2 diabetes (T2D) were enrolled. Plasma fibrinogen concentrations and peripheral white blood cells were measured, and MLR was calculated, and the associations of FC-MLR with DR and severity of disease were assessed. Regression analysis and receiver operating characteristic (ROC) curves were performed to evaluate the risk factors and predictive power of FC-MLR for DR and severity of disease, respectively. DR patients showed higher fibrinogen concentrations and a higher MLR than did T2D patients without complications (P<0.01); Moreover, DR patients in proliferative stage also showed higher fibrinogen concentrations and a higher MLR than did those in non-proliferative stage (P<0.01). FC-MLR was closely associated with occurrence and severity of DR (P<0.01), and was an independent risk factor for them (*OR*=6.123, 95%CI: 3.122-17.102; and 7.932, 95%CI: 4.315-16.671, respectively; P<0.001). The predictive sensitivity and specificity for DR and severity of disease were 0.86 and 0.68, and 0.85 and 0.73, respectively. The study suggests that FC-MLR may be used as a predictor for the risk and progression of diabetic retinopathy.

## Introduction

AS one of the most common causes, diabetic retinopathy (DR) contributes to 2.6% and 1.9% of blindness and visual impairment in worldwide 1. However, yearly examination of the retina or retinal photography is often delayed until after severe damage even visual impairment has occurred 2. Therefore, early identification of the microvascular complications risk provides an opportunity to stop or delay disease onset 3, which would be more valuable to improve the prognosis of T2D patients. Studies have shown that many biomarkers can reflect the presence of microvascular damage 4,5, and they are also associated with the risk of retinopathy 6,7,8. However, to our knowledge, most of those markers are not the convenient and practical ones for the prediction, diagnosis, and prognostic assessment of DR. Thus, there are some clinical limitations for them, and it would be of great clinical significance to explore some more valuable markers for early screening and assessing the disease progression of patients with DR.

Increasing evidence points to inflammation as a critical contributor to the development of DR 9, and many inflammatory markers are associated with increased risk of DR 10. Therefore, inflammation-related markers would play important roles in prediction and disease assessment of DR. Liu *et al.* has reported that WBC subtypes were closely associated with inflammatory state of DR patients 11, which indicates that peripheral blood parameters of patients would be of great value for DR diagnosis and treatment. Monocyte-lymphocytes ratio (MLR), a novel inflammatory marker, plays an important role in prediction and prognosis of some tumors and cardiovascular diseases 12,13. It has been recognized that tumors and cardiovascular diseases as well as DR are inflammation-related diseases. However, the associations of MLR with DR remains unclear. On the other hand, fibrinogen is an acute-phase reactant glycoprotein mainly synthesized by hepatocytes 14. Furthermore, plasma fibrinogen is important in the regulation of inflammatory response 15, and plasma fibrinogen levels are associated with increased inflammation 10. Our previous studies have revealed that plasma fibrinogen is a powerful predictor for diabetic nephropathy in patients with T2D 16, and combined plasma fibrinogen concentrations and MLR (FC-MLR) can be used as a powerful prognostic index of ovarian cancer 18. Therefore, fibrinogen may play a combined role with MLR in inflammatory diseases. Although the associations of FC-MLR with DR is unknown, we speculated that both plasma fibrinogen concentrations and MLR may play important roles in development and progression of DR, and there would be a valuable significance of combined FC-MLR in DR patients. However, the association of FC-MLR with DR is unknown. Thus, the aim of this study was to explore the clinical and predictive significance of FC-MLR in DR patients.

## Materials and methods

### Patients' population

A total of 307 patients with T2D were finally enrolled in the study. All patients were the outpatients or inpatients from the Departments of Endocrinology, Zhejiang Provincial People's Hospital, China, between October 2015 and December 2016. T2D patients without diabetes-related complications and those with retinopathy only before daily treatment were included in the study. All patients with T2D were diagnosed according to the criteria in the 2013 Diabetes Guideline from the China Diabetes Association 18. Patients who exhibited the following characteristics were included: 1) typical symptoms of diabetes (polyuria, polydipsia, unexplained weight loss); 2) random blood glucose level ≥11.1 mmol/l; 3) fasting plasma glucose (FPG) level ≥7.0 mmol/l or 2-h post-challenge glucose level in oral glucose tolerance test ≥11.1 mmol/l. Any atypical symptoms were confirmed on a different day. The following exclusion criteria was used: 1) diabetes-related complications except DR; 2) primary liver and kidney dysfunctions; 3) cardiovascular and cerebrovascular diseases; 4) malignancies; 5) acute inflammation or infections; 6) postoperative status; and 7) incomplete data. Retinopathy of diabetic patient was assessed using digital retinal photographs. According to severity of disease, DR is divided into two stages: non-proliferative diabetic retinopathy (NPDR, an early stage) and proliferative diabetic retinopathy (PDR, an advanced stage). NPDR and PDR are characterized as follows, respectively: during NPDR stage, retinal pathologies including microaneurysms, hemorrhages and hard exudates can be detected by fundus photograph, and during PDR stage, the patients may experience severe vision impairment when the new abnormal vessels bleed into the vitreous (vitreous hemorrhage) or when tractional retinal detachment is present 19. The basic evaluation before daily diabetic-related treatment included clinical assessment (disease course: time since diagnosis of diabetes, risk factors, data of physical examination, and clinical and biologic data), and the major laboratory tests. T2D patients were divided into two groups including T2D without diabetic-related complications group (212 patients) and T2D with retinopathy group (95 patients). At the same time, 101 healthy controls (age, sex, and race-matched) were included in the study. The control subjects came from the healthy management center at the hospital, and had not taken any drugs in the 2 weeks before sample collection. Informed consent was obtained from the controls and patients, and this study was approved by the Hospital's Ethics Committee.

### Laboratory assay

Samples were collected and prepared as the description previously 20, 21. In brief, venous blood samples were collected in sodium citrate- or EDTA-K_2_-containing and anticoagulant-free vaccutainer tubes (Becton Dickinson, MountainView, CA, USA) after an overnight fast before treatment, respectively. Prothrombin time (PT), international normalized rario (INR), activated partial thromboplstin time (aPTT), thrombin time (TT), fibrinogen (Fbg), and D-dimer (D-D), were measured by a coagulation analyzer (CS-5100, Japan Sysmex) and the commercial reagents (Siemens Healthcare Diagnostics Products GmbH) within four hours after sample collection, respectively. Total WBC, monocyte, and lymphocyte levels were measured by an automatic hematological analyzer and commercially available reagents (BC-6900; Mindray Inc., Shenzhen, China) in one hour after sample collection. Sera were obtained from the blood samples without anticoagulants by centrifugation at 1500×*g* for 10 min at room temperature. Subsequently, the levels of other biochemical indexes including plasma high-sensitive C-reactive protein, serum Cystatin C, Thyroid stimulating hormone, Fasting serum glucose, Peptide-C, Glycosylated hemoglobin, Serum high density lipoprotein, Total cholesterol, and Albumin were measured. At the same time, the biological and clinical data of the patients and controls (age, sex, and body mass index for all subjects; T2D-related and pathological data before in-hospital treatment for all patients) were collected and reviewed. The analyzers were well calibrated, and an internal quality analysis was well performed. All experiments were performed in accordance with relevant guidelines and regulations.

### Statistical analysis

Data were initially tested for distribution normality by the Kolmogorov-Smirnov test, and normally distributed data were presented as mean ± standard deviation. One-way analysis of variance was used to analyze the differences among among controls, T2D and DR patients, and differences between two groups were subsequently analyzed by the Student-Newman-Keuls (SNK) test. Samples with normal and non-normal data distributions were analyzed by Student's *t*-test and the Mann-Whitney *U*-test, respectively. The Chi-square test was used for categorical variables (clinical and biological characteristics and patient percent in different FC-MLR groups). Multivariate logistic regression analyses were performed to calculate the odds ratio and 95% confidence interval (95%CI) for DR. A receiver-operating characteristic (ROC) curve was constructed, and the area under the curve (AUC) was calculated to evaluate the predictive power of the independent risk factors. All statistical analyses were performed using the SPSS 20.0 statistical package (SPSS, Chicago IL, USA). A value of *P*<0.05 was considered statistically significant.

## Results

### Clinical and biological characteristics of patients with type 2 diabetes

In this study, 307 consecutive patients with confirmed diagnosis of T2D were enrolled. The present study initially investigated the basic characteristics of the patients. The median age at diagnosis was 62 years old (range: 37-85 to 85 years), and male patients constituted majority of the group (n=201, 65%). Overall, about 65% of the patients showed elevated plasma fibrinogen concentrations and MLR (*P*<0.05). The detailed comparisons of the basic clinical and biological characteristics are presented **in Table 1**.

### Clinical and biological parameters of T2D patients and controls

Furthermore, the study also assessed the differences between the basic clinical and biological parameter levels among the control subjects and the patients. There were increases in D-D, CRP, and WBC subtypes levels except for a decreased lymphocyte count in T2D patients without complications (P<0.01). However, DR patients showed significantly higher levels of D-D, CRP, and monocyte counts, but a decreasing of lymphocyte and PLT count than did the patients without complications (P<0.01 for all variables). Other parameters did not exhibit significant differences between the groups. The detailed data were presented in **Table 2**.

### Associations of plasma fibrinogen concentrations with clinical characteristics of T2D patients

**Figure 1:**
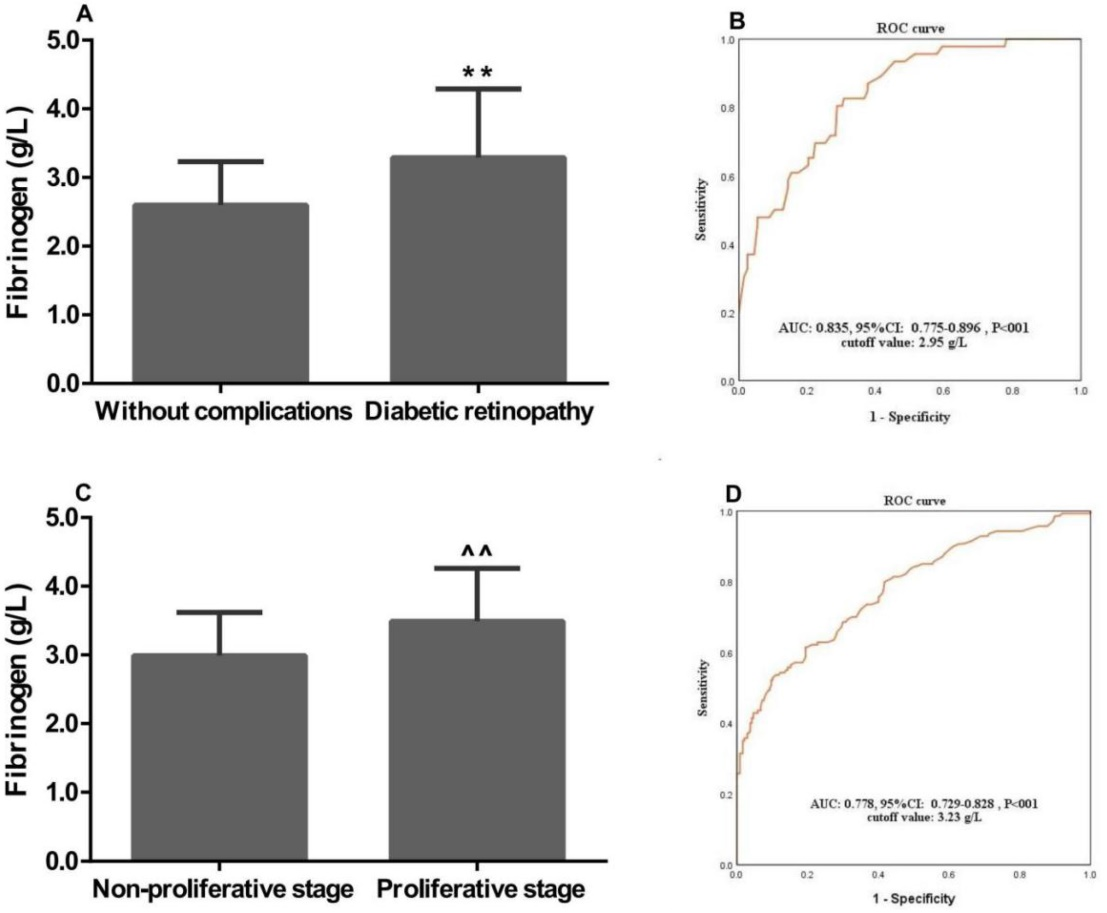
** Associations of plasma fibrinogen concentrations with the clinical characteristics of patients with T2D and DR.** Fibrinogen concentrations was significantly associated with (A) diabetic retinopathy, and (C) proliferative stage; ROC curve for discriminating between patients with (B) DR and T2D without complications, and (D) non-proliferative and proliferative stage; ROC: Receiver-operating characteristic; T2D: type 2 diabetes; DR: diabetic retinopathy; AUC: area under the curve. **, ^^, P<0.01 vs. “Without complications” and “Non-proliferative stage” patients by the* t*-test, respectively.

In this study, the associations of plasma fibrinogen concentrations with the clinical characteristics of T2D patients were assessed. The mean plasma fibrinogen concentrations were 3.29±1.00 and 2.60±0.63 g/L in patients with DR and without complications, respectively. Plasma fibrinogen concentrations were significantly associated with the occurrence of DR (P<0.01, **Figure 1A**). Further study showed that plasma fibrinogen concentrations were significantly higher in patients with proliferative stage than those with non-proliferative stage (3.49±0.77 g/L *vs.* 2.99±0.63 g/L, P<0.001; **Figure 1C**). The ROC curve analysis indicated plasma fibrinogen had a high AUC [AUC: 0.835; 95% confidence interval (CI): 0.775-0.896; P<0.01] for discriminating between patients with DR and T2D without complications. When the optimal cutoff value was set at 2.95 g/L, the sensitivity and specificity were 0.78 and 0.71, respectively (**Figure 1B**). Moreever, the ROC curve also demonstrated a high AUC (AUC: 0.778; 95% CI: 0.729-0.828; P<0.01) of plasma fibrinogen concentrations, with an optimal cutoff value of 3.23 g/L for discriminating between the DR patients with proliferative and non-proliferative stage. And based on that cutoff value, the sensitivity and specificity were 0.82 and 0.75, respectively (**Figure 1D**). For the subsequent study, patients were assigned to normal and high fibrinogen groups according to the cutoff values.

### Associations of the MLR with the clinical characteristics of T2D patients

**Figure 2:**
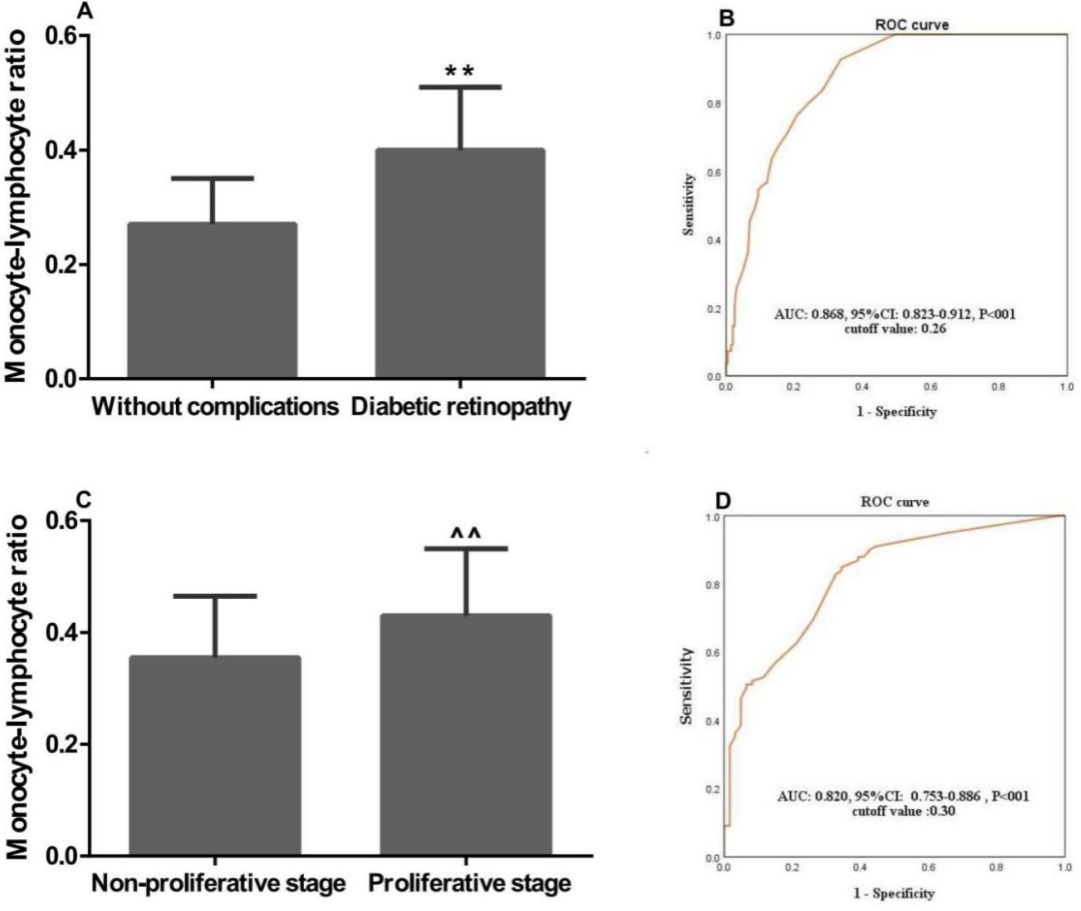
** Monocyte-lympocyte ratio and the clinical characteristics of patients with T2D and DR.** MLR was significantly associated with (A) diabetic retinopathy, and (C) proliferative stage; ROC curve for discriminating between patients with (B) DR and T2D without complications, and (D) non-proliferative and proliferative stage; ROC: Receiver-operating characteristic; T2D: type 2 diabetes; DR: diabetic retinopathy; AUC: area under the curve. **, ^^, P<0.01 vs. “Without complications” and “Non-proliferative stage” patients by the t-test, respectively.

We further investigated the associations of MLR with the clinical characteristics of T2D patients. In patients with DR, MLR was significantly higher than that in patients with T2D without complications (0.40±0.11 *vs.* 0.27±0.08, P<0.01; **Figure 2A**). Furthermore, MLR in DR patients with proliferative stage was remarkably higher than that in patients with non-proliferative stage (0.430±0.12 vs. 0.355±0.11, P<0.01; **Figure 2C**). The ROC curve analysis showed a high AUC (AUC: 0.868; 95%CI: 0.823-0.912; P<0.01) of MLR for discriminating between the patients with DR and T2D without complications. And the sensitivity and specificity were 0.81 and 0.71, respectively, when the optimal cutoff value was set at 0.26 (**Figure 2B**). At the same time, we also found MLR had a high AUC (AUC: 0.820; 95% CI: 0.753-0.8886; P<0.01) with an optimal cutoff value of 0.30 for discriminating between the DR patients with proliferative and non-proliferative stage, and the sensitivity and specificity were 0.85 and 0.73, respectively (**Figure 2D**). For the following study, patients were divided to two groups including a normal and a high MLR according to the cutoff values.

### Associations of FC-MLR with DR and disease condition

**Figure 3:**
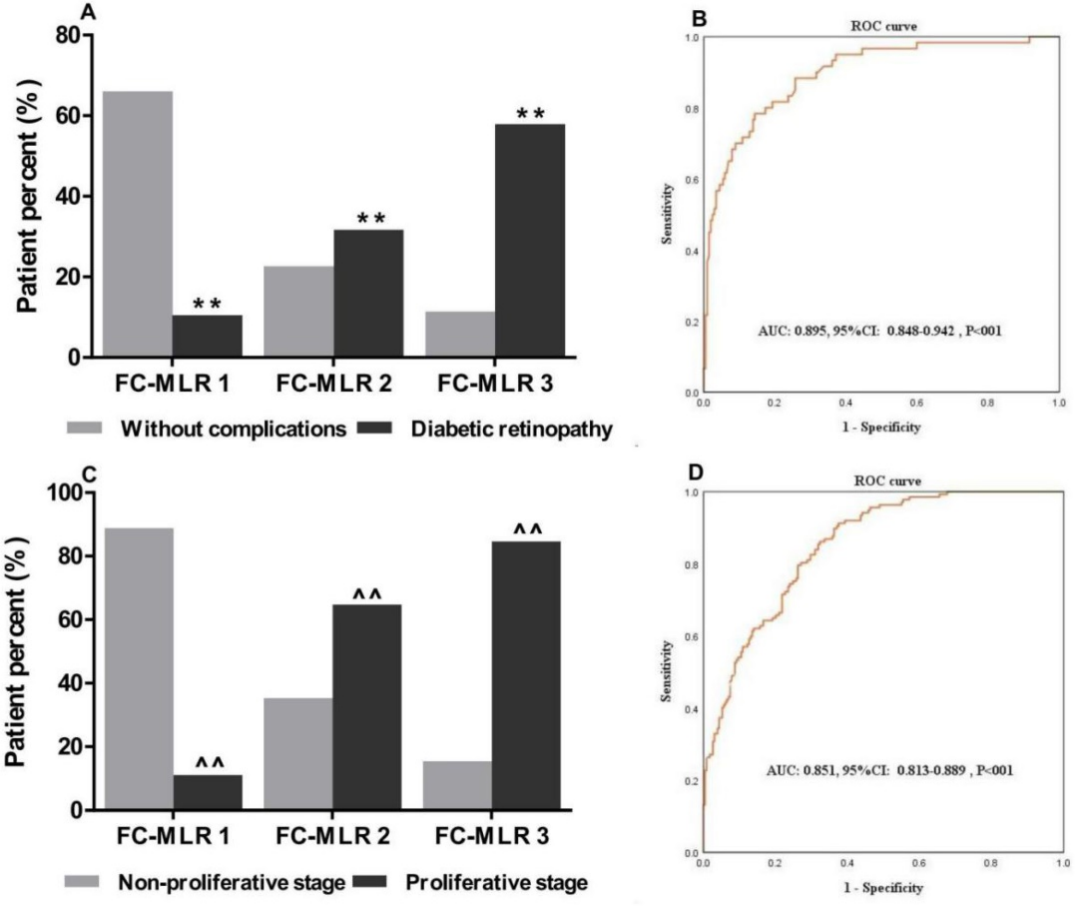
** Associations of combined plasma fibrinogen concentrations and MLR with the clinical characteristics of patients with T2D and DR.** FC-MLR was significantly associated with (A) diabetic retinopathy, and (C) proliferative stage; ROC curve for discriminating between the patients with (B) DR and T2D without complications, and (D) non-proliferative and proliferative stage; ROC: Receiver-operating characteristic; T2D: type 2 diabetes; DR: diabetic retinopathy; AUC: area under the curve. **, ^^, P<0.01 vs. “Without complications” and “Non-proliferative stage” patients by the *Chi-square* test, respectively.

Given that plasma fibrinogen concentrations and MLR were closely associated with the occurrence and severity of DR, we simultaneously investigated the associations of plasma fibrinogen concentrations combined MLR with DR and disease condition. We first divided the patients into three groups (group FC-MLR 1: no abnormality; group FC-MLR 2: either high fibrinogen concentrations or a high MLR; group FC-MLR 3: both high plasma fibrinogen concentrations and a high MLR) according to their cutoff values. We found that there was a significantly higher percent of DR patient than that of T2D patients without complications in group FC-MLR 3, and the percent of DR patient in group FC-MLR 1 was significantly lower than that in group FC-MLR 3 (57.9% *vs.* 11.4% and 10.5% *vs.* 66.0%, respectively, both P<0.01;** Figure 3A**). Moreover, there was a gradual increasing of percent of the DR patient with proliferative stage from group FC-MLR 1 to 3 (11.1%, 64.7%, and 84.6%, respectively, P<0.01;** Figure 3C**). The ROC curve analysis further showed a high AUC (AUC: 0.895; 95%CI: 0.848-0.942; P<0.01) of FC-MLR for discriminating between the patients with DR and T2D without complications, and the sensitivity and specificity were 0.86 and 0.68 based on the optimal cutoff values of fibrinogen and MLR, respectively (**Figure 3B**). At the same time, the study also exhibited a high AUC (AUC: 0.852; 95% CI: 0.813-0.889; P<0.01) of FC-MLR for discriminating between DR patients with proliferative and non-proliferative stage, which the sensitivity and specificity were 0.85 and 0.73 according to the cutoff values of fibrinogen and MLR, respectively (**Figure 3D**).

### Associations of plasma fibrinogen, the MLR, and FC-MLR with the risk and progression of DR

We finally investigated the associations of plasma fibrinogen, MLR, and FC-MLR as well as HbA1c and disease course, recognized risk factors for DR, with the risk and progression of DR. For this purpose, a multivariate analysis was performed. The analysis demonstrated that plasma Fbg concentrations, MLR and FC-MLR were independently correlated with the risk and progression of DR, and all were the independent predictors for them (P<0.001, respectively). The detailed data were presented in **Table 3**.

## Discussion

The present study measured plasma Fbg concentrations and MLR in 307 patients with T2D. We also assessed the associations of Fbg, MLR and FC-MLR with DR and its severity. Within our knowledge, this might be the first study to investigate the clinical significance of FC-MLR in patients with DR. Two of the major findings in the study were that FC-MLR was significantly correlated with risk and severity of DR, and it might be used as a predictor for the occurrence and progression of DR.

Fibrinogen is an acute-phase reactant protein that is released in response to infection or systemic inflammation 23. Moreover, it has been well established that total fibrinogen is elevated during inflammation 24. Many studies have revealed that patients with diabetic microvascular complications would have high plasma fibrinogen concentrations, and plasma fibrinogen was significantly increased with severity of diabetic retinopathy 25,26,27. In the present study, higher Fbg concentrations were found in DR patient than that without complications, which indicated that the elevation of Fbg level is associated with DR. It has been well known that fibrinogen may induce the synthesis of interleukin-6, an inflammatory mediator, or interact with leukocytes 23. Therefore, hyperfibrinogenemia may occur secondary to chronic inflammation that occurs in response to the occurrence and progression of DR, which may reversely cause the increasing of plasma Fbg concentrations, and measurement of plasma Fbg will be useful in discriminating patients from DR and T2D without complications. Our study further showed that plasma Fbg had a powerful diagnostic significance with a high AUC and medium sensitivity in discriminating between patients with DR and T2D without complications. Thus, the findings suggest that plasma Fbg also is a useful predictor for the occurrence of DR. The study further showed that there was a higher plasma Fbg concentration in DR patient with proliferative stage than that with non-proliferative stage, and plasma Fbg also had a powerful diagnostic significance with a high AUC and high sensitivity according to the optimal cutoff value in discriminating between DR patients with proliferative and non-proliferative stage. Our findings revealed that elevated plasma Fbg concentrations were closely associated with high proliferative stage of DR, and Fbg will be a valuable predictor for the progression of DR. Therefore, plasma Fbg can be used as a predictor for the occurrence and progression of DR.

It is known that DR are closely associated with chronic inflammatory responses 28. A systemic inflammatory response causes variations in the balance of circulating white blood cell constituents 29. Grossmann* et al.* showed that WBCs, granulocytes, and monocytes gradually increased from normoglycemic subjects to subjects with diabetes, whereas the lymphocyte concentration was stable despite the disease progression in diabetes 30. And Luo *et al.* reported that NLR and PLR could be recommended as diagnostic biomarkers for DR 31. Moreover, Ji *et al. also* reported that monocyte to lymphocyte ratio (MLR) or lymphocyte to monocyte ratio (LMR) could mirror the circulating immune status of the host 32. Thus, these findings suggest that systemic inflammatory response can induce an increased monocyte and decreased lymphocyte in pathogenesis of DR, and lymphopenia and an elevated monocyte count would stand for a microenvironment surrogate marker of the occurrence and progression of DR. Therefore, MLR might be a good reflection of different clinical conditions in T2D and DR patients. In the present study, we found that DR patients had remarkably higher MLR than those without complications, which indicates that a high MLR in DR patient may be resulted from an increased number of monocytes and a decreased number of lymphocytes because of an enhanced inflammatory response and a reduced immune function. Therefore, the findings suggest that MLR is closely associated with the risk of DR. Concerning the increasing of the MLR, we speculate that it may be associated with the production of the pro-inflammatory chemokines such as IL-6, TNF-α, IL-1β, and MCP-1 which play major role in the recruitment and activation of monocytes and leukocytes and the subsequent inflammatory responses in DR patients 9. Thus, observing the changes in monocytes and lymphocytes of peripheral blood is sure to have clinical significance in discriminating patients from DR and T2D without complications. Additionally, our findings also showed that MLR had a high AUC and high sensitivity in discriminating DR and T2D without complications, indicating that MLR is a powerful predictor for the occurrence of DR. And the study further demonstrated a significantly higher MLR in DR patient with proliferative stage than that with non-proliferative stage, and MLR also had a high AUC and high sensitivity in discriminating DR patients between proliferative and non-proliferative stage. Our findings were not consistent with that of the previous study 33, which may be because of regional differences between the included subjects. In the study, our findings reveal a close association of elevated MLR with enhanced severity of DR, indicating that MLR is a powerful predictor for the progression of DR. Therefore, our findings suggest that MLR measurement will be valuable for predicting the occurrence and progression of DR.

The main finding of the study was that combination of plasma FC-MLR was a novel index for evaluating the occurrence and progression of DR. Multivariate regression analyses indicated that plasma Fbg, MLR and FC-MLR were independently associated with the risk and progression of DR, but FC-MLR showed a more powerful clinical significance in discriminating between DR and T2D without complications as well as advanced (proliferative stage) and early (non-proliferative stage) DR than single Fbg or MLR. To further assess the predictive power of FC-MLR, the patients with T2D and DR were divided into three groups according to the cutoff values of plasma Fbg concentrations and MLR. We found that patients with both high fibrinogen concentrations and a high MLR had a higher percent of DR patient than did those without high fibrinogen concentrations and a high MLR. And we also found that DR patients with high fibrinogen concentrations and a high MLR were closely associated with a higher patient percent in proliferative stage than did other DR patients. Furthermore, the ROC curve of FC-MLR showed a greater predictive power with high sensitivity and specificity in discriminating between DR and T2D without complications as well as advanced and early DR than did Fbg concentrations or MLR. Therefore, our findings suggest that plasma fibrinogen concentrations combined MLR before treatment would be useful for assessing the risk and progression of DR in patients with T2D. This information can be used to prevent and treat diabetic microvascular complications, which may be practical for the improvement of patient prognosis. FC-MLR is a simple index which is obtained from Fbg concentrations and calculated MLR derived from conventional blood analysis. Consequently, FC-MLR can be used as a cost-effective, convenient, practical, and powerful predictor for DR and a prognostic index in patients with T2D.

There may be some limitations of the present study. In our study, we only reviewed 95 DR patients in one hospital, and there probably were some subclinical DR patients or patients with other complications which were not diagnosed before treatment. On the other hand, the predictive models with the cutoff values and AUCs of ROC curves in the present study did not be externally validated in different study population and other centers. Therefore, the results may have been influenced by the small sample size, the fact that we did not perform different population and multicentric study, and the mismatched patients. However, the present study also reveals a notable significance of combined plasma fibrinogen concentrations and MLR for assessing the occurrence and progression of diabetic retinopathy.

## Conclusions

This study suggests that combined plasma fibrinogen concentrations and MLR is closely associated with the occurrence and severity of diabetic retinopthy and FC-MLR may be a useful predictor for the risk and progression of diabetic retinopthy. However, we should further emphasize that our conclusion could not be used in patients with acute infection because of a close association of MLR with some viral infections.

## Figures and Tables

**Table 1:** Clinical and biological characteristics of patients with type 2 diabetes

	Number	%	*P* value
n	307	100	
**Age, years**			
Median (range)	62 (37-85)		
-60	160	52	
60+	147	48	>0.05
**Gender**			
Male	201	65	
Female	106	35	<0.05
**Complications**			
Without	212	69	
Retinopathy	95	31	<0.01
**DR Stage**			
Non-proliferative stage	34	36	
Proliferative stage	61	64	<0.01
Systolic pressure			
≥140 mmHg	60	20	
<140 mmHg	247	80	<0.01
**eGFR**			
≥90 ml/min	234	76	
<90 ml/min	73	24	<0.01
**UAE**			
≥300 mg/24 h	104	34	
<300 mg/24 h	203	66	<0.05
***Thyroid stimulating hormone***			
Normal	210	68	
Elevated	97	32	<0.01
**Fasting serum glucose**			
Normal	43	14	
Elevated	264	86	<0.01
**Peptide-C**			
Normal	181	59	
Elevated	126	41	>0.05
**Glycosylated hemoglobin A1c**			
<7.0%	234	76	
≥7.0%	73	34	<0.01
**Serum high density lipoprotein level**			
Normal	174	57	
Decreased	133	43	>0.05
**Serum cholesterol**			
Normal	199	65	
Elevated	108	35	<0.05
**Serum albumin**			
Normal	256	83	
Decreased	51	17	<0.01
**Plasma fibrinogen**			
Normal	201	65	
Elevated	106	35	<0.05
**MLR**			
Normal	204	66	
Elevated	103	34	<0.05

†. Normal, elevated or decreased criteria was based on the upper or lower limit of reference interval of variables, respectively. eGFR: estimated glomerular filtration rate; UAE: urinary albumin excretion; MLR: monocyte-lymphocyte ratio; *P* value: comparisons of patient percentage between the two groups by Chi-square test.

**Table 2:** Comparisons of clinical and biological parameters of T2D patients and controls

Variables	Controls	T2D patients without complications	DR patients	*P*
n	101	212	95	
Gender (M/F)	68/33	129/83	60/35	>0.05
Age, years^Δ^	56 (33-79)	57 (31-82)	67 (35-87)	>0.05
PT, s	11.3±1.4	11.0±1.5	11.2±1.6	>0.05
INR	0.98±0.3	0.95±0.2	0.96±0.4	>0.05
aPTT, s	26.5±3.8	25.2±3.2	26.0±4.7	>0.05
PLT, 10^9^/L	211 (89-379)	201 (55-477)	189 (75-664)**	<0.01
D-D, mg/L	0.20 (0.05~0.48)	0.44 (0.05-4.01)*	0.53 (0.06-6.31)**	<0.01
TT, s	17.6±1.4	16.8±1.3	17.4±1.2	>0.05
WBC (×10^9^/L)	5.81±1.65	7.15±1.88*	6.95±2.11*	<0.01
CRP (mg/L)	1.25 (0.20~6.34)	4.55(0.20~15.41)*	8.91 (0.20~23.22)**	<0.01
Neutrophil (×10^9^/L)	3.78±1.03	4.55±1.22*	4.53±1.25*	<0.01
Monocyte (×10^9^/L)	0.30±0.09	0.50±0.10*	0.66±0.12**	<0.01
Lymphocyte(×10^9^/L)	1.45±0.21	1.85±0.30*	1.65±0.24**	<0.01

†. Data were presented as mean and standard deviation, and the variables with (^Δ^) were present as median and the range. T2D: type 2 diabetes mellitus; DR:diabetic retinopathy; M: males; F: females; PT: prothrombin time; INR: international normalized ratio; aPTT: activated partial thromboplastin time; PLT: platelet count; D-D: D-dimer; TT: thrombin time; WBC: white blood cell; CRP: C-reactive protein.* P* value: comparisons of the three groups by one way analysis of variance. *P<0.01: compared with Controls, and **P<0.001: compared with T2D without complications analyzed by SNK-test.

**Table 3:** Multivariate Logistic regression analysis in T2D and DR patients

Variables	For risk of DR			For progression of DR		
	*OR*	95%CI	*P*	*OR*	95%CI	*P*
Course of disease	13.771	7.425-22.893	0.000	16.012	8.645-33.778	0.000
Fbg	4.516	2.886-13.655	0.000	4.289	2.401-10.430	0.000
MLR	5.302	2.925-15.201	0.000	6.272	3.689-15.312	0.000
FC-MLR	6.123	3.122-17.102	0.000	7.932	4.315-16.671	0.000

†. T2D: type 2 diabetes; DR: diabetic retinopathy; Fbg: fibrinogen; MLR: monocyte-lymphocyte ratio; FC-MLR: fibrinogen concentrations combined the monocyte-lymphocyte ratio; *OR*: odds ratio. CI: confidence interval.

## Abbreviations

T2D: type 2 diabetes
DR: diabetic retinopathy
FC: fibrinogen concentrations
PDR: proliferative diabetic retinopathy
NPDR: non-proliferative diabetic retinopathy
FPG: fast plasma glucose
PT: prothrombin time
INR: international normalized ratio
aPTT: activated partial thromboplastin time
PLT: platelet count
D-D: D-dimer
TT: thrombin time
WBC: white blood cell
CRP: C-reactive protein
eGFR: estimated glomerular filtration rate
UAE: urinary albumin excretion
ROC: receiver operating characteristic
AUC: area under curve
OR: odds ratio
CI: confidence interval
